# Exploring the Prognostic, Mutational and Therapeutic Potential of ANXA2 in Ovarian Cancer via Multi-Omics and In Silico Approach

**DOI:** 10.3390/biology15070523

**Published:** 2026-03-25

**Authors:** Prithvi Singh, Joyeeta Talukdar, Hajed Obaid A. Alharbi, Wanian M. Alwanian, Indrakant Kumar Singh, Arshad Husain Rahmani

**Affiliations:** 1 Centre for Interdisciplinary Research in Basic Sciences, Jamia Millia Islamia, New Delhi 110025, India; prithvi.mastermind@gmail.com; 2 Department of Biochemistry, All India Institute of Medical Sciences, New Delhi 110029, India; 3 Department of Medical Laboratories, College of Applied Medical Sciences, Qassim University, Buraydah 51452, Saudi Arabia; 4 Dr. B.R. Ambedkar Center for Biomedical Research, University of Delhi, New Delhi 110007, India

**Keywords:** ovarian cancer, Apolipoproteins, Annexins, molecular docking, ADMET properties, MD simulation

## Abstract

Background: Among gynecological malignancies, ovarian cancer (OC) remains a leading cause of mortality worldwide, often characterized by the highest fatality-to-case ratio due to its asymptomatic progression and late-stage detection. Despite substantial investigation, the root cause of disease development and pathology remains unknown. Early detection is critical for improving OC prognosis. Unfortunately, because of the lack of identifiable symptoms in the early stages, the disease is frequently detected late. As a result, regular check-ups, being aware of risk factors, and paying attention to unusual symptoms can all help discover OC early. Apolipoproteins (APOs) and Annexins (ANXs) have recently been linked to OC. Aim: We conducted a cutting-edge bioinformatics investigation to find novel therapeutic targets and precise biomarkers linked to OC against APO and ANX. Methods: We started by compiling the ANX and APO families via HUGO Gene Nomenclature Committee (HGNC) homepage. Next, we accessed GEPIA2 to compare the relative messenger RNA (mRNA) expression levels of all ANX and APO family members across the cancer genome atlas (TCGA)-OC cohort and matched normal and GTEx data. Prognostic analysis of all significantly expressed ANXs and APOs was performed via Kaplan–Meier (KM) plotter. cBioPortal was used for mutational analysis of prognostic ANXs and APOs. Finally, we ran functional enrichment, molecular docking, and molecular dynamics (MD) simulation analyses. Results: Overall, the results suggest that *ANXA2* and its related genetic changes represent potential focal points for precision oncology, offering a computational rationale for the development of target-driven therapeutic interventions in OC. Conclusions: Molecular docking and MD simulation analyses identified curcumin as a potential inhibitor of ANXA2, demonstrating stable binding affinity and structural conservation throughout the simulation period. These computational findings characterize curcumin as a promising candidate for targeting ANXA2 in OC, warranting further experimental validation to confirm its therapeutic efficacy.

## Simple Summary

Ovarian cancer is particularly deadly because it is often detected too late. This study used advanced bioinformatics to search for better diagnostic tools and treatments, focusing on two protein families: Apolipoproteins (APOs) and Annexins (ANXs). By analyzing genetic data from TCGA, we identified ANXA2 as a significant marker for the disease. Using computer simulations to test potential drug interactions, we discovered that curcumin—a natural compound—effectively binds to and stabilizes against the ANXA2 protein. This suggests that curcumin could potentially block the protein’s harmful activity. While these computational results are a promising step toward precision medicine, the researchers emphasize that further laboratory testing is needed to confirm curcumin’s effectiveness as a targeted therapy for ovarian cancer patients.

## 1. Introduction

OC is one of the leading causes of death in women, and the prognosis is heavily impacted by the disease’s stage [1]. OC is usually identified in its late stages since it is largely asymptomatic in its early stages. GI is one of the hallmarks of OC [2]. OC is classified into numerous categories, the most frequent of which is epithelial OC. GC tumors and stromal tumors are two less prevalent forms. OC can strike at any age, although it is more common in postmenopausal women. OC is caused by several risk factors, some of which are listed as follows: family history, genetic mutations (such as *BRCA1* and *BRCA2*), age, and a history of breast, colorectal, or endometrial cancer may increase the risk of OC.

A physical examination, imaging tests and blood tests (including the CA-125 blood test, though it is not specific to OC) are commonly used to diagnose OC. Surgery to extract and evaluate the ovarian tissue is frequently used to get a definite diagnosis. Early detection is critical for improving OC prognosis. Regretfully, because the disease’s early-stage symptoms are not yet sufficiently distinguishable, it is frequently detected late. As a result, regular check-ups, being aware of risk factors, and paying attention to unusual symptoms can all help discover OC early [3]. APOs and ANXs have recently been linked to a variety of malignancies, including breast [4] and OC [5,6]. The overexpression of *ANXA2* and its critical role in the progression of epithelial OC have been well-documented, specifically correlating high expression levels with poor patient survival outcomes [7,8].

APOs are proteins that join forces with lipids (fats) to create lipoproteins, which are essential for the bloodstream’s transit and metabolism of lipids [9]. While APOs are primarily associated with lipid transport and cholesterol metabolism, emerging research suggests that they may also have implications in cancer development and progression [10,11,12]. The link between cholesterol metabolism and cancer is an area of active research [13,14]. Lipids play a crucial role in cell membrane structure, and alterations in lipid metabolism can influence cancer cell growth and survival. Some cancer cells have an increased demand for cholesterol to support their rapid growth, and alterations in lipoprotein metabolism may contribute to cancer development [14,15]. For example, alterations in circulating levels of certain APOs may be associated with the presence or progression of certain cancers. The use of APOs as biomarkers is an evolving area of research and may have diagnostic or prognostic implications in the future [10,16].

On the other hand, ANXs are a family of calcium-dependent phospholipid-binding proteins that are involved in several cellular processes, such as membrane trafficking, apoptosis (PCD), inflammation, and the regulation of cellular signaling pathways [17,18]. Studies have linked ANXs to both normal cellular processes and the initiation and spread of different types of cancer. Apoptosis, a PCD mechanism necessary for preserving tissue homeostasis, may involve ANXs [19]. Dysregulation of apoptosis is a hallmark of cancer, and certain ANXs may influence whether cells undergo PCD or survive. ANXs have also been implicated in cell migration and invasion, critical processes in cancer metastasis. ANXs such as *ANXA2* have been associated with promoting cell migration and invasion in some cancer types [20]. ANXs also contribute to TA, the creation of new blood vessels, which is crucial for tumor growth and progression. *ANXA2*, for example, has been linked to TA in prostate cancers [1], RCC [2], TNBC [3], esophageal cancer [4], etc. Some ANXs have been explored as potential diagnostic and prognostic markers for certain cancers [21], e.g., liver cancer [5], pancreatic cancer [6], colorectal cancer [9], etc. Given their involvement in OC, targeting signaling pathways of ANXs and APOs has emerged as a potential therapeutic strategy [22]. Preclinical and clinical studies are exploring approaches to inhibit ANXs and APOs or block their receptors to suppress tumor growth, TA, and metastasis, and enhance the effectiveness of existing treatments. The co-investigation of APOs and ANXs is biologically significant as they represent two pillars of the lipid-membrane interface. While the individual roles of APOs in metabolic transport and ANXs in structural scaffolding are recognized, their combined influence as a synchronized ‘lipid–membrane axis’ in OC progression remains poorly defined. We structured this study to test the hypothesis that the deadliest phenotypes of OC are driven by a synergy between the metabolic lipid availability provided by APOs and the structural membrane-remodeling capacity of ANXs. Consequently, our study design moves beyond simple expression profiling; we utilized a multi-omics filtration process to isolate family members that serve as both functional drivers and significant prognostic indicators of poor patient survival. While APOs orchestrate the metabolic availability of cholesterol and lipids necessary for rapid tumor growth, ANXs translate these lipid signals into structural membrane changes that facilitate invasion and metastasis. Together, their dysregulation in OC supports critical hallmarks such as TA, metabolic reprogramming, and evasion of apoptosis.

Turmeric’s natural ingredient, curcumin, has drawn significant attention due to its potential medical uses. By blocking several inflammatory pathways and lowering the synthesis of pro-inflammatory chemicals, curcumin demonstrates potent anti-inflammatory effects [23]. It can suppress the activity of TFs such as NF-κB, which play a key role in the inflammatory response in OC [24]. Curcumin has demonstrated the ability to modulate multiple signaling pathways, thereby showing promise in the management of inflammatory diseases such as RA [25], IBD [26], and CIDs [27]. Its anti-cancer potential, particularly in OC, has been widely explored [28]. The selection of curcumin as a therapeutic lead is grounded in established evidence demonstrating its capacity to modulate the protein–lipid interface. Prior investigations have shown that curcumin targets multiple enzymes in the ROS metabolic pathway to suppress tumor growth and acts as a synergistic regulator of the ANXA2, EGFR, and MMP signaling axes. While these studies documented curcumin’s influence on the ANXA2 pathway at the expression level, the physical stability of their direct interaction remains a critical research gap. This motivated our study to move beyond broad pathway modulation and investigate the structural feasibility of curcumin as a direct physical inhibitor of the ANXA2-binding domain.

Curcumin exerts its anti-tumor effects through various mechanisms [29], including inhibition of tumor growth, induction of apoptosis (PCD) in cancer cells [30], and suppression of TA. Moreover, it has been studied for its capacity to enhance the efficacy of chemotherapy and radiotherapy by sensitizing cancer cells to these treatments. As a natural phenolic compound, curcumin continues to gain attention for its therapeutic role in oncology research [31]. Over the years, it has been employed as a treatment strategy in various cancers, including LC [32] and OC [28], rather than being currently employed as a standard clinical treatment strategy. Beyond these broad anti-tumor effects, we selected curcumin as a candidate inhibitor because of its documented ability to modulate the protein–lipid interface. Given that ANXs function as phospholipid-binding scaffolds and APOs act as essential lipid-carrying mediators, we hypothesized that curcumin could physically disrupt the structural binding domains required for their activity. This investigation seeks to bridge the gap between curcumin’s known anti-metastatic properties and its potential to directly inhibit the ANXA2 and APOC2 proteins in OC phenotypes.

It may be advantageous to target a particular protein, and bioinformatics has made this feasible [33]. The transition from genomic identification to therapeutic targeting requires a precise understanding of structural compatibility. We adopted an integrated in silico pipeline that combines DGE with MD simulations to bridge the gap between biomarker identification and functional inhibition. This methodology allows for the simulation of physical ‘anchoring’ within the protein’s binding pocket, providing a structural rationale for inhibition that traditional expression analysis alone cannot provide. One potentially effective therapeutic approach to managing OC is to inhibit ANXs and APOs. It is crucial to remember that the roles played by ANXs and APOs in OC are complicated and might differ depending on the OC’s molecular features and subtypes. To gain a deeper understanding of the precise mechanisms and possible therapeutic implications of APOs and ANXs in the development and treatment of OC, more study is being conducted. In this study, we hypothesized that the dysregulation of specific ANX and APO family members drives OC progression and facilitates metastasis through altered membrane signaling. Consequently, we investigated curcumin as a potential therapeutic inhibitor that could directly target these prognostic markers. By integrating differential expression analysis, survival modeling, and MD simulations, we aim to characterize the structural and functional potential of the ANXA2–curcumin complex as a novel therapeutic strategy for OC.

While *ANXA2* is a documented driver of epithelial OC progression, its activity is intrinsically linked to the availability of lipids at the membrane interface. Consequently, we initially investigated both the ANX and APO families to identify the most potent prognostic targets within this lipid–membrane axis. This dual-family approach served as a systematic selection filter, allowing us to objectively prioritize candidates with the highest differential expression and the strongest correlation to poor OS.

## 2. Materials and Methods

### 2.1. ANX and APO Family Expression Analysis Across OC Cohort

HGNC (https://www.genenames.org/, accessed on 10 September 2025) was accessed for the compilation of all ANX and APO family members. For evaluating the relative mRNA expression levels of all ANX and APO family members across TCGA-OC cohort and matched normal and GTEx data, GEPIA2 web-based tool [34,35] (http://gepia2.cancer-pku.cn/, accessed on 20 September 2025) was queried. Statistically significant (i.e., *p*-value <0.05 and $\left|{log}_{2}FC\right|>1$) ANX and APO family members were picked for further analysis.

### 2.2. Prognostic and Mutational Analyses of ANX and APO Family Members Across OC Cohort

To investigate the prognostic potential of ANX and APO family members having significant expression, we accessed the KM plotter web-based tool [36] (https://kmplot.com/analysis/, accessed on 30 September 2025). Patients were bifurcated into high and low expression groups based on the median mRNA expression of the target gene to provide a robust and unbiased OS comparison. KM plots showing significant (i.e., log-rank *p*-value <0.05) OS of these family members across TCGA-OC patient samples were reported. To investigate the mutations and putative CNAs of prognostically significant ANX and APO family members, we queried the cBioPortal web-based tool [37,38] (https://www.cbioportal.org/, accessed on 25 October 2025).

### 2.3. Pathway and GO Enrichment Analyses

Top 10 significant (i.e., *p*-value <0.05) GO terms and pathways corresponding to prognostic and genetically altered ANX and APO family members were collected via accessing Enrichr web-based tool [39,40] (https://maayanlab.cloud/Enrichr/, accessed on 30 October 2025). Reactome, GO-BP, GO-MF, and GO-CC libraries were used for compiling pathway and GO terms data.

### 2.4. Preparation of Protein and Ligand

After downloading the crystal structures of APOC2 (PDB ID: 1SOH) and ANXA2 protein (PDB ID: 2HYU) from the RCSB PDB [41] (https://www.rcsb.org/, accessed on 11 November 2025), we imported them into the PyMol visualizer tool version 2.6 [42] and found that the structure was complete. After that, the model was opened in PyMol, and to clear the way for ANXA2 and APOC2 binding sites or allosteric sites, waters and extra ligand entries were eliminated. Curcumin, a small molecule (ID: 969516), was obtained via PubChem (https://pubchem.ncbi.nlm.nih.gov/, accessed on 13 November 2025). It complies with the RO5 [43], which specifies that molecules must have a molar refractivity of 40–130, a molecular mass of less than 500 Dalton, a LogP of less than 5, and H-bond donors and acceptors of less than 5.

### 2.5. Molecular Docking

Upon identifying ANXA2 and APOC2 as significant prognostic markers with potential roles in tumor growth and TA, we sought to identify a therapeutic lead capable of modulating their activity. Curcumin, a natural phenolic compound with documented efficacy in inhibiting ovarian cancer cell proliferation and suppressing inflammatory signaling pathways, was selected as a candidate inhibitor for molecular docking. This selection was further justified by curcumin’s known ability to disrupt protein–lipid interactions, making it a suitable candidate for targeting the membrane-binding domains of the ANX and APO families.

Molecular docking is widely recognized as a crucial strategy in drug discovery due to its ability to significantly reduce both the time and cost involved in identifying and developing potential therapeutic candidates. After creating the receptor and ligand files in the pdbqt format, we carried out molecular docking. After docking the compound with the best binding affinities, InstaDock version 1.0 [44] was used to obtain the log and out file. Then, Pymol and Discovery Studio visualizer tools were employed for interaction analysis.

We used the PyMol program to explore the crystal structures of the ANXA2 and APOC2 receptor proteins (PDB IDs: 2hyu and 1soh), which we found to be complete with no missing aa residues. The structures were taken from the RCSB PDB. A key component of in silico drug development is the ability of molecular docking studies to predict the exact binding structures of tiny molecules that act as ligands to the right binding of the target site. Considering this, the goal of our study was to investigate the molecular docking of curcumin for possible inhibition of ANXA2 and APOC2, two crucial proteins targeted in cancer. Moreover, molecular docking was carried out using the InstaDock following the synthesis of proteins and small compounds. This approach produces data on the binding energy and intermolecular distance between the binding residues in addition to the receptor–ligand interaction. Using automated independent software, InstaDock produces energy files for the interactive hits.

#### 2.5.1. Calculation of pKi

The G parameter was used to calculate the pKi, or negative decimal logarithm of inhibition constants [45], using the formula below.

$$∆G\times 1000=RT(ln{Ki}_{pred})$$

$${Ki}_{pred}=e^{\left(∆G\times 1000/RT\right)}$$

(1) $$pKi=-log{(Ki}_{pred})$$

where T is the room temperature (298.15 Kelvin), R is the gas constant (1.987 cal/(mol·K)), and ${Ki}_{pred}$ is the expected inhibitory constant. Factor of 1000 is used to convert ∆G from kcal/mol to cal/mol to maintain unit consistency with R.

#### 2.5.2. ADMET

The initial requirement in the design of new medications is safety, which was approved for pharmaceuticals and evaluated using ADMET to determine efficacy. The curcumin compound’s ADMET properties were evaluated using the ADMETlab 2.0 (https://admetmesh.scbdd.com/) and SwissADME (http://www.swissadme.ch/index.php, accessed on 16 November 2025) platforms. Because both websites support it, SMILEs were collected from the databases linked with the molecules to access the databases.

### 2.6. MD Simulations

MD simulations predict each atom’s movement within molecular systems over time, utilizing a standard model of physics governing interatomic interactions [46]. Before starting the MD simulation, there are steps which should be executed sequentially: preparation of the protein file, conversion of PDB to Gromacs format and generation of topology, creation of box dimensions, solvation of the protein, addition of ions, and energy minimization. Gromacs version 2020.6 was utilized, and the charmm36-jul2022 FF was employed. The topology of the ligand was generated using the CGenFF program and cgenff_charmm2gmx_py3_nx2.py script. Each system was centered in a cubic box with a distance of 10 Å from the edges, and the SPC/E water model was applied. Equilibration was conducted in two stages: isothermal/isochoric (NVT) for 100 ps at 300 K, followed by isobaric (NPT) stabilization for 100 ps after temperature equilibration. Subsequently, a 100 ns MD simulation was executed, and the resulting trajectory files were analyzed using GROMACS built-in tools.

## 3. Results

### 3.1. ANX and APO Family Expression Analysis Across OC Cohort

**Figure 1:**
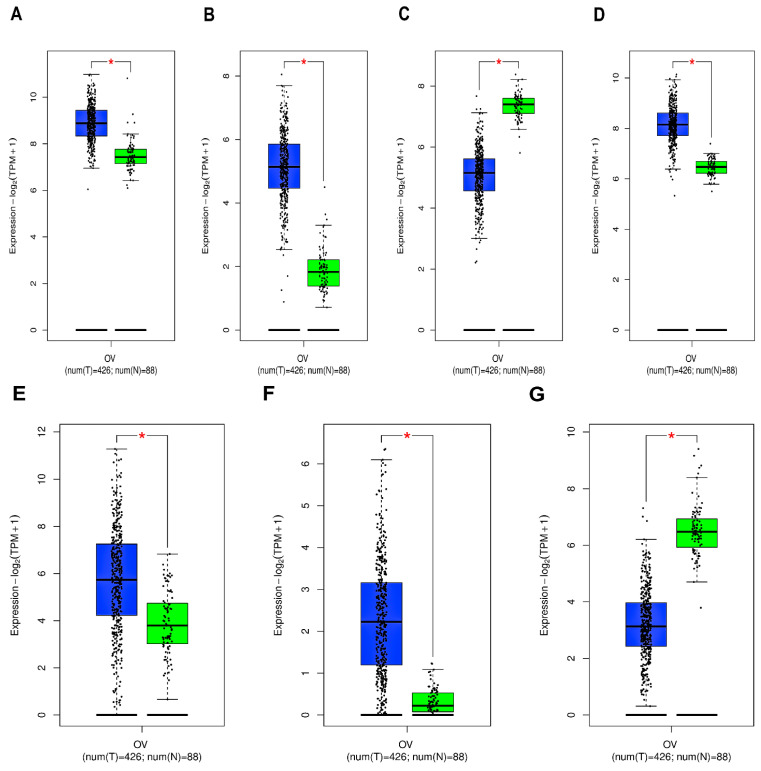
Box-and-whisker plots exhibiting the mRNA expression levels of (**A**) ANXA2, (**B**) ANXA3, (**C**) ANXA6, (**D**) ANXA11, (**E**) APOA1, (**F**) APOC2, (**G**) APOD across tumor and normal samples. Green- and blue-colored box areas imply normal and tumor patient samples. * *p*-value <0.05.

*ANXA2*, *ANXA3*, *ANXA11*, *APOA1* and *APOC2* expression levels were significantly upregulated while *ANXA6* and *APOD* expressions were significantly downregulated in tumor samples in comparison to normals as indicated by the box-and-whisker plots in Figure 1.

### 3.2. Prognostic and Mutational Analyses of ANX and APO Family Members Across OC Cohort

**Figure 2:**
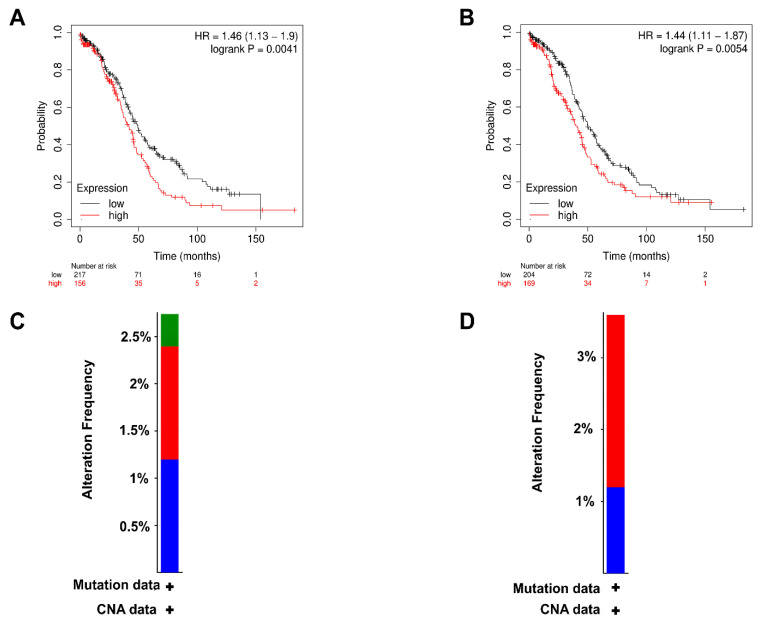
KM plots demonstrate OS of (**A**) ANXA2 and (**B**) APOC2 across the TCGA-OC cohort. Barplots demonstrate alteration frequencies of (**C**) ANXA2 and (**D**) APOC2 across the TCGA-OC cohort. Red, blue, and green colored bars signify amplifications, deep deletions, and missense mutations.

Using KM plotter, prognostic analysis was performed on *ANXA2*, *ANXA3*, *ANXA6*, *ANXA11*, *APOA1*, *APOC2*, *APOD* across TCGA-OC cohort. However, only *ANXA2* and *APOC2* reported significant log-rank *p*-values between higher and lower expression groups. Additionally, survival trends for ANXA2 and APOC2 mirrored their expression levels. KM plots as shown in Figure 2A,B revealed significantly poor OS across 374 OC patients when mRNA expression levels of *ANXA2* and *APOC2* were high. cBioPortal was used to evaluate the specific genetic modifications associated with *ANXA2* and *APOC2* across the OC dataset (TCGA, Firehose legacy) comprising 600 primary tumor samples. The analysis of cancer types elaborates the overall frequency of changes in these genes as shown in Figure 2C,D. The cumulative alteration frequency for the prognostic pair *ANXA2* and *APOC2* was 6% (36/600) patient samples. When examining individual genomic changes, *APOC2* and *ANXA2* exhibited sequence-level mutation frequencies of 4% and ~2.7%, respectively. Specifically, for *ANXA2*, we observed 1.17% (7/600) amplifications, 0.33% (2/600 cases) missense mutations, and 1.17% (7/600) deep deletions. In the case of *APOC2*, the frequencies were 2.33% (14/600) for amplifications and 1.17% (7/600) for deep deletions. These genetic alterations, though low in frequency, coincide with the significantly poor OS observed in patients with high mRNA expression of these genes.

### 3.3. Pathway and GO Term Enrichment Analyses

**Figure 3:**
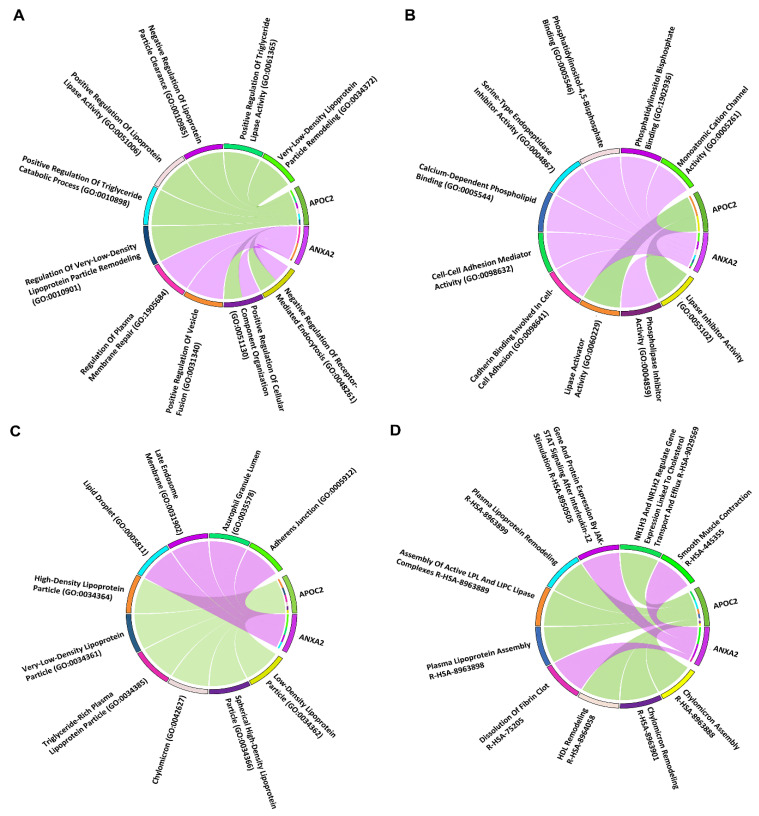
Chord plots exhibiting the ANXA2 and APOC2 linkage with top 10 significant (**A**) GO-BP, (**B**) GO-MF, (**C**) GO-CC, (**D**) pathway terms via undirected colored edges.

Link of *ANXA2* and *APOC2* with top 10 significant pathways, GO-BP, GO-MF, GO-CC terms were presented via chord plots in Figure 3. The significant pathway, GO-BP, GO-MF and GO-CC terms were chylomicron assembly (*p*-value $=8.9\times 10^{-4}$), negative regulation of receptor-mediated endocytosis (*p*-value $=1.6\times 10^{-6}$), lipase inhibitor activity (*p*-value $=8.9\times 10^{-4}$) and low-density lipoprotein particle (*p*-value $=5.9\times 10^{-4}$).

### 3.4. Molecular Docking Analysis

**Figure 4:**
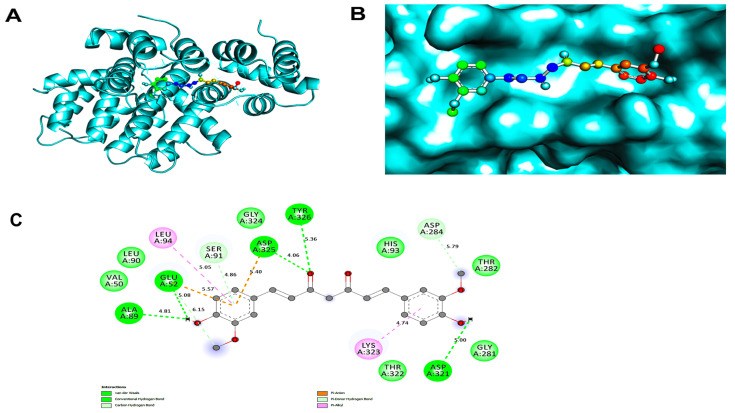
(**A**) Cartoon view of ANXA2–curcumin complex, (**B**) Magnified surface view of ANXA2–curcumin complex, (**C**) Two-dimensional view of residues interacting between ANXA2–curcumin complex.

Using formula Equation (1), we calculated the pKi of the hits. Curcumin interacted with the ANXA2 and APOC2 proteins, with energy values −5.8 kcal/mol and −7.0 kcal/mol respectively (Table 1). Curcumin interacts with strong affinity showing its binding pockets (Figure 4). The compound established critical bonds such as H-bonds with the residues TYR326, ASP325, GLU52, ALA89, and ASP329 (Figure 4C). Beyond the primary H-bond network, the structural integrity of the ANXA2–curcumin complex is further stabilized by an array of non-covalent hydrophobic interactions, including van der Waals forces, alkyl/pi-alkyl contacts, and pi-sigma interactions. (Figure 4C). APOC2 and curcumin interactions were also analyzed; none of the H-bonds formed between APOC2 and curcumin (Figure S1). Consequently, ANXA2 and curcumin were further studied for MD simulation.

#### ADMET Properties

Evaluating ADMET properties is important for the identification and progression of drug-like molecules. Compounds that meet physicochemical and ADMET criteria have a higher probability of advancing to clinical application. The physicochemical and ADMET profiles of curcumin were assessed using SwissADME and ADMETlab 2.0 (Table 2). The compound complied with the Lipinski, Ghose, Veber, and BMS rules, and no PAINS alerts were observed. Additionally, various other parameters supported its drug-like potential. There are six H-bond acceptors and two H-bond donors, a consensus log P of 3.03, a molecular weight (g/mol) of 368.38, and a TPSA of $93.06\;{Å}^{2}$.

### 3.5. MD Simulation Analysis

#### 3.5.1. Structural Deviations and Compactness

RMSD is a key parameter commonly used to assess the structural stability of a protein and its similarity to the experimentally determined conformation. The unbound ANXA2 protein exhibited an average RMSD of 0.15 nm, indicating a stable structure. Upon binding with curcumin, minor conformational shifts were observed in the active site, with the average RMSD increasing slightly to 0.16 nm (Table 3). The ANXA2–curcumin complex showed initial deviations below 0.2 nm, which stabilized throughout the simulation, reaching a maximum RMSD of approximately 0.25 nm. While localized RMSF peaked at approximately 0.4 nm in flexible regions, the overall structural variation remained within a narrow range indicative of a stable complex (Figure S2A).

The local structure’s flexibility determines the oscillations surrounding the point of stability, which are not random. By graphing the RMSF of the ANXA2 following drug binding as a function of residue number, the mean variation in all residues during the simulation was determined (Table 3). Residual aberrations are present throughout the ANXA2 structure, as indicated by the RMSF figure. It was demonstrated that these residual fluctuations were lowest when ANXA2 and curcumin were coupled together, and they were also lowest when ANXA2 and curcumin were bound to different protein regions (Figure S2B). Overall, no variation was observed. The probability distribution plots of RMSD and RMSF (Figure S2C,D) further quantify these oscillations around the point of stability, confirming that no major structural perturbations occurred upon curcumin binding.

A measure of a protein’s secondary structure volume called $\;R_{g}$ has been used to provide light on how stable a protein is in a biological system [28]. Because of the less compact packing, a protein must have a larger $R_{g}$. The average $R_{g}$ of ANXA2 and complexed with curcumin were 2.1 (Table 3). The $R_{g}$ plot shows that there is no difference in the packing of ANXA2 after ligand binding (Figure S3A,C).

While APOC2 exhibited a superior binding affinity, we prioritized the ANXA2–curcumin complex for further MD analysis due to the presence of specific H-bonding interactions. Mechanistically, H-bonds provide the directional specificity and structural ‘anchoring’ required for effective protein inhibition. Unlike non-specific hydrophobic interactions which can drive a high docking score but result in higher ligand mobility, the H-bonds formed with TYR326 and ASP325 in ANXA2 ensure a stable residence time. This ‘lock-and-key’ fit effectively traps the ligand within the binding pocket, significantly increasing the probability of functional disruption compared to a purely hydrophobic association.

#### 3.5.2. SASA

The average SASA values for ANXA2 ($159\pm 1.2\;{nm}^{2}$) and its complex ($158\pm 1.4\;{nm}^{2}$) showed no significant deviation, suggesting that the protein’s solvent exposure remained constant upon ligand binding (Table 3). The SASA plot indicates that the overall SASA values of ANXA2 and its curcumin complex vary little (Figure S3B,D).

#### 3.5.3. H-Bond Analysis

**Figure 5:**
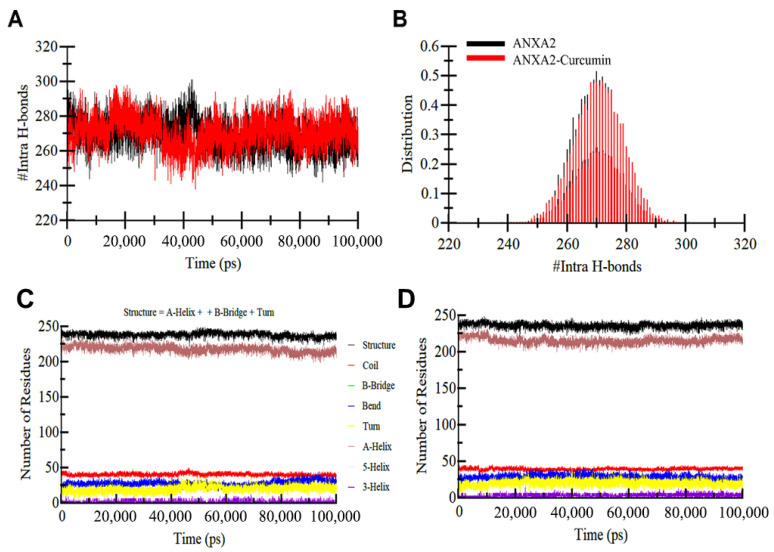
(**A**) The changes in H-bonds formed within ANXA2 before and after curcumin binding, (**B**) Distribution plot of intramolecular H-bonds. Secondary structure change in ANXA2 (**A**) before and (**B**) after curcumin binding. (**C**) secondary structure profile of the unbound ANXA2 protein throughout 100 ns simulation, (**D**) secondary structure profile of the ANXA2-curcumin complex throughout 100 ns simulation.

Directionality and specificity of interactions are made possible by H-bonding between chemicals and proteins, which is essential for molecular recognition. Intramolecular H-bond analysis (apo: 269±5; complex 270±4) demonstrated a preserved internal network. Figure 5A,B confirm that these values represent stable equilibrium states rather than transient fluctuations, providing a quantitative basis for the structural stability of the ANXA2–curcumin complex.

#### 3.5.4. Changes in Secondary Structure

The secondary structure content of ANXA2 was monitored throughout the 100 ns simulation to assess structural integrity upon ligand binding. A consistent mean of 77% of the residues participated in secondary structure formation in both the apo and complex states, with the proportion of coils remaining constant at 13%. These data, alongside the stable α-helix content (~70–71%), demonstrate that ANXA2 maintains its native fold and does not undergo unfolding or significant conformational degradation upon interaction with curcumin (Table 4, Figure 5C,D). This may suggest that ANXA2 partially unfolds upon binding curcumin.

## 4. Discussion

Our findings identified ANXA2 as a primary prognostic marker and therapeutic target in OC. This is clinically significant because ANXA2 acts as a key scaffold for membrane remodeling and signaling complexes that drive the invasive phenotype of high-grade serous ovarian carcinomas. The strong correlation observed between high ANXA2 expression and poor OS suggests that this protein facilitates tumor cell migration and metastasis through its interactions at the lipid–membrane interface. The functional significance of the ANXA2–curcumin interaction lies in the potential disruption of the protein’s membrane-binding capability. ANXA2 mediates cancer progression primarily through its role as a calcium-dependent phospholipid-binding protein, facilitating the assembly of signaling complexes at the plasma membrane that drive EMT. Our MD simulations suggest that curcumin occupies a critical structural pocket, potentially interfering with the coordination of calcium ions or the orientation of the phospholipid-binding loops. By disrupting this interaction, curcumin may antagonize ANXA2-mediated cell migration and invasion, effectively preventing the protein from performing its scaffolding function required for tumor metastasis. Although *APOC2* exhibited significant pathways related to lipase inhibition, it lacked the structural stability required for inhibition, thus confirming *ANXA2* as the primary therapeutic focus of this study.

By identifying curcumin as a stable structural inhibitor, we provide a potential molecular strategy to disrupt these ANXA2-mediated pathways. Unlike broad-spectrum chemotherapeutics, the specific anchoring of curcumin to the ANXA2 binding pocket validated by our stable 0.25 nm RMSD trajectory offers a mechanism to potentially suppress metastasis by interfering with the protein’s native phospholipid-binding capacity.

Analyzing GED can assist in uncovering potential treatment targets by identifying genes that have different expression patterns in disease conditions. An aberrant DGE in a disease state may be crucial to the onset and course of the illness. For instance, the overexpression or underexpression of genes may promote CP, the ability to avoid PCD, or the capacity to invade other tissues, all of which may aid in the onset of cancer. Additionally, it is possible to diagnose cancer or forecast the possibility of a certain event using the differential expression levels of specific genes.

OC’s increased death rate highlights the need for new biomarkers, therapeutic agents, and molecular therapeutic targets to be identified and further discovered for the development of early detection and efficient therapy. To develop an effective therapy, it is crucial to identify gene targets associated with cancer characteristics. Currently, in silico methods are used on a larger scale to identify the essential regulating gene [3,47,48,49,50]. The quantification and distinction of gene expression levels between normal and malignant samples are made possible through the analysis of gene expression profiles from several databases.

In this study, we have gathered the lists of all members of the APO and ANX families by accessing the HGNC homepage. The relative mRNA expression levels of each member of the ANX and APO families throughout the TCGA-OC cohort and matched normal and GTEx data were then compared using GEPIA2. After that, we evaluated the predictive potential of ANX and APO family members exhibiting substantial expression using the KM plotter. Significant OS of these family members across TCGA-OC patient samples was displayed in published KM plots. *ANXA2* and *APOC2* were finalized prognostic biomarkers which were subjected to mutational analysis via cBioPortal. Next, Enrichr was used to compile the top 10 significant pathway and GO terms that were explicitly related to *ANXA2* and *APOC2*.

OC is largely influenced by the tumor’s growth and mutated genes that act as a potential control mechanism. Our research also reports the maximum number of genes being involved in various pathways, BPs, MFs, and CCs. The most significant pathway was chylomicron assembly in which *APOC2* was present. Also, the most significant GO-BP term was negative regulation of receptor-mediated endocytosis in which both *APOC2* and *ANAX2* were present. Endocytosis rates are frequently elevated in cancer cells to meet their high metabolic needs. Encouraging cell development and proliferation involves internalizing nutrients and GFs from the extracellular environment [51]. Further, the most significant GO-MF term was lipase inhibitor activity in which *APOC2* was present. Inhibiting lipase activity may theoretically affect the availability of fatty acids for cancer cell metabolism. Lipids, including fatty acids, are important for energy storage as well as crucial roles in cellular signaling pathways. Changes in lipid metabolism may influence signaling pathways that contribute to cancer progression [52]. Cancer cells frequently exhibit altered energy metabolism, including an increased demand for nutrients, including fats, to support their rapid growth and proliferation. Clinical studies and research are currently being conducted with an emphasis on immune-based treatments, which are increasingly used to treat OC.

Recently, researchers have concentrated on the roles of various types of APOs [10] and ANXs in cancer, autophagy, OS, and drug resistance. APOs and ANXs’ potential use as biomarkers for cancer diagnosis and prognosis brought up, as an inquiry into their potential as therapeutic targets for cancer treatment. Through serial examination of gene expression, ApoE has recently been identified as a putative tumor-associated marker in OC [53]. The overexpression of ApoE was observed in a variety of OC cell lines and tissue [10]. Similarly, ANX protein has been linked to several critical cancer-related processes, such as cell division, apoptosis, chemosensitivity, metastasis, and invasion [54]. Changes in the expression levels of specific ANXs may be associated with the presence, progression, or prognosis of certain cancer types. The roles of ANXs in cancer are complex and can vary depending on the specific ANX and the type of cancer [19]. Different ANXs may exhibit diverse functions in different cellular contexts and cancer types [55,56]. Because of their involvement in various cellular processes related to cancer, ANXs have been considered as potential therapeutic targets [21]. Modulating the activity of specific ANXs may have implications for cancer treatment. Therefore, the primary goal of this suggested inquiry was to identify and validate important regulatory genes associated with APOs and ANXs that are linked to OC phenotypes using in silico methods.

*ANXA2* contributes to the organization and dynamics of the membrane by binding to phospholipids in a calcium-dependent way. It participates in several cellular functions, such as exocytosis, organization, and membrane trafficking [57,58]. The growth and proliferation of cells have been linked to *ANXA2*, and cancer has been linked to its dysregulation. Numerous malignancies have been shown to overexpress *ANXA2*, and research is being done to determine whether this contributes to the disease’s advancement. It has been linked to the invasion, migration, and metastasis of cancer cells. The development and aggressiveness of tumors may be influenced by the overexpression of *ANXA2* in cancer [55,59]. *ANXA2* has been investigated as a possible biomarker for specific cancers due to its participation in the disease, and its findings could impact targeted treatments in the future [21,55].

Humans have 22 genes in the APO gene family, including *APOA1*, *APOA2*, and so on [10,60]. Gene mutations can change the roles of APOs during synthesis, which can result in the development of many disorders such as hyperlipidemia, atherosclerosis, and CAD. Additionally, because of their overexpression or lack thereof, APOs are linked to the development and spread of several malignancies [10]. Research shows how distinct APO expressions manifest in various patient malignancies [61].

Further, we carried out molecular docking analysis of ANXA2 receptor protein. The ANXA2 protein was employed in this research to perform an in silico approach against curcumin. Curcumin, derived from Curcuma longa, exhibits inhibitory effects on cyclin D, cyclin E, CDK2, CDK4, and CDK6 activity, as reported in the literature [62]. Recent experimental evidence supports this hypothesis, demonstrating that curcumin targets multiple enzymes involved in the ROS metabolic pathway to suppress tumor cell growth [63]. Furthermore, curcumin extracts have been shown to synergistically regulate the signaling pathways of ANXA2, EGFR, and MMPs [64]. Our in silico analysis extends these findings by demonstrating that curcumin not only influences *ANXA2* at the expression level but may also physically interact with the ANXA2 protein at a structural level with favorable binding affinity.

Our findings provide a computational basis for the potential inhibition of ANXA2 by curcumin in OC. The stability of the ANXA2–curcumin complex over a 100 ns trajectory suggests that curcumin may act as a competitive or allosteric inhibitor by occupying key binding residues. While this study establishes the structural feasibility of this interaction, further experimental validations including binding affinity assays and functional knockdown studies are essential to confirm that this binding event directly translates to the suppression of ANXA2-mediated cell migration and metastasis in OC phenotypes.

Molecular docking is a well-established structure-based computational approach frequently employed in drug discovery and development. It facilitates the identification of novel therapeutic agents and enables the prediction of ligand–target interactions at the molecular level. In this study, curcumin was virtually screened against the ANXA2 protein to evaluate its binding potential. Additionally, its physicochemical and ADMET profiles were analyzed using the SwissADME and ADMETlab 2.0 platforms to assess its drug-likeness and pharmacokinetic behavior.

After the compound with the best binding affinities was docked, InstaDock was utilized to obtain the log and out file. The ANXA2 protein and curcumin interacted with energy values −5.8 kcal/mol (Table 1). Curcumin binds with strong affinity showing their binding pockets are presented in Figure 4. With the residues, the molecule formed crucial bonds like H-bonds TYR326, ASP325, GLU52, ALA89, and ASP329. Other interactions were also formed and the distance between bonds was also calculated.

The physicochemical and ADMET properties of curcumin were evaluated using the SwissADME and ADMETlab 2.0 platforms (Table 2). The compound satisfied multiple drug-likeness criteria, including Lipinski, Ghose, Veber, and BMS rules, and no PAINS alerts were detected. Curcumin displayed favorable drug-like features with a molecular weight of 368.38 g/mol, a consensus LogP value of 3.03, six H-bond acceptors, two H-bond donors, and a TPSA of $93.06{\;Å}^{2}$. Further assessment of its pharmacokinetic suitability through ADMET profiling done by MD simulations was conducted using GROMACS version 2020.6.

In the absence of any ligand, the ANXA2 protein exhibited an average RMSD of 0.15 nm, indicating structural stability. Upon binding with curcumin, slight conformational shifts were observed, with the average RMSD increasing marginally to 0.16 nm. The ANXA2–curcumin complex showed initial deviations below 0.2 nm, which gradually increased during the simulation, reaching a maximum RMSD of approximately 0.25 nm. These results suggest minimal structural perturbation upon ligand interaction. All things considered, we can say that ligand binding varies somewhat (Figure S2A). Plotting the RMSF of the ANXA2 after drug binding as a function of residue number yielded the average variance of all residues during the simulation. Residual aberrations are seen throughout the ANXA2 structure, as indicated by the RMSF figure. The RMSF plot showed the residual differences in different regions of the ANXA2 protein structure. The maximum of these residual variations was noted at 0.4 after the binding of curcumin. Ultimately, no variation was discovered. Because of the looser packing, a protein’s $R_{g}$ needs to be larger [43]. ANXA2 and its complex with curcumin had an average $R_{g}$ of 2.1 (Figure S3A). The SASA analysis revealed no significant difference in total SASA between the unbound ANXA2 protein and its curcumin complex (Figure S3B), suggesting stable solvent exposure upon ligand binding. H-bond analysis was carried out to assess molecular interactions between ANXA2 and curcumin. The results indicated that curcumin formed three stable hydrogen bonds within the active site of ANXA2, with minimal fluctuation throughout the simulation.

Additionally, we examined changes in the protein’s secondary structure upon ligand binding. The average number of residues participating in secondary structural elements remained largely unchanged between the apo form and the ligand-bound complex, indicating structural integrity was maintained. The MD trajectory data were further analyzed to quantify intramolecular H-bonds in ANXA2 over time. This comparison between the apo and ligand-bound states demonstrated consistent H-bond formation, suggesting that curcumin binding did not disrupt the internal H-bonding network and may contribute to maintaining the protein’s overall structural stability. During the simulation, the PDF for intramolecular H-bonds showed a fair amount of consistency in all systems. Overall, we can say that the ANXA2–curcumin complex was relatively stable during MD-Simulation; however, further experimental validations are needed to clarify the findings.

This study employed an integrated computational pipeline utilizing bioinformatics, molecular docking, and 100 ns MD simulations to identify and characterize the potential inhibition of ANXA2 by curcumin. However, it is important to note that these findings are based on predictive models and lack direct experimental validation in OC cell lines or animal models. Therefore, the predicted ANXA2–curcumin interactions and their subsequent effect on tumor progression require functional validation through cell-based assays and in vivo studies. Such experimental proof is a prerequisite before clinical translation or drug development strategies targeting the ANX/APO axis can be considered. Future research will focus on quantifying the binding affinity through biophysical techniques and evaluating the impact of curcumin on ANXA2-mediated migration in high-grade serous OC phenotypes.

## 5. Conclusions

In conclusion, this in silico study identified ANXA2 and APOC2 as significant prognostic markers associated with poor OS in OC. Through an integrated pipeline of molecular docking and 100 ns MD simulations, we computationally predicted curcumin as a potential therapeutic inhibitor capable of forming a stable complex with the ANXA2 protein. However, as this research is purely computational, these findings require experimental validation through cell-based assays and in vivo studies before clinical consideration can be addressed. This study establishes a refined structural foundation to guide the future experimental development of targeted therapies for OC.

It is crucial to understand that *ANXA2* plays a complex role in OC and that the outcomes will depend on the situation, how it interacts with other signaling pathways, and other elements of the TME. More research is required to comprehend the ANXA2–curcumin function and possible therapeutic implications in OC completely. Research into the precise processes and therapeutic possibilities of targeting ANXA2–curcumin is now continuing.

## Figures and Tables

**Table 1:** Curcumin interacted with the ANXA2 and APOC2 proteins, with energy values.

SI. No.	Protein	Compound	Binding Affinity (kcal/Mol)	pKi
1.	ANXA2	Curcumin	−5.8	4.25
2.	APOC2	Curcumin	−7.0	5.13

**Table 2:** ADMET properties of curcumin.

SI. No.	Compound ID	Phytochemical	Absorption	Distribution	Metabolism	Excretion	Toxicity
			GI Absorption	P-gp Substrate	CYP2C19 Inhibitor/subst.	OCT2 Substrate	hERG I Inhibitor
**1.**	969516	Curcumin	High	NO	NO	NO	NO

**Table 3:** A generalized overview of all the parameters of MD simulations.

System	RMSD (nm)	RMSF (nm)	$R_{g}$ (nm)	SASA (nm^2^)	Intramolecular H-Bonds
ANXA2	0.15	0.09	2.1	159	269
ANXA2–Curcumin	0.16	0.09	2.1	158	270

**Table 4:** The percentage of residues that are normally involved in the formation of the protein’s secondary structure.

System	Structure	Coil	B-Bridge	Bend	Turn	A-Helix	5-Helix	3-Helix
ANXA2	0.77	0.13	0.00	0.09	0.07	0.71	0.00	0.00
ANXA2–Curcumin	0.77	0.13	0.00	0.09	0.07	0.70	0.00	0.01

## Data Availability

The original contributions presented in this study are included in the article/Supplementary Material. Further inquiries can be directed to the corresponding authors.

## Supplementary Materials

The following supporting information can be downloaded at: https://www.mdpi.com/article/10.3390/biology15070523/s1. Figure S1. Representation of molecular docking result. (A) cartoon view of APOC2 in-complex with Curcumin. (B) Surface view of APOC2 in-complex with Curcumin. (C) 2D view of interacting residues of APOC2 with Curcumin. Figure S2. (A) RMSD plot exhibiting ANXA2 and ANXA2-curcumin complex deviation over 100 ns simulation. (B) RMSF plot exhibiting ANXA2 and ANXA2-curcumin complex fluctuation over 100 ns simulation. Probability distribution plot of (C) RMSD and (D) RMSF. Figure S3. (A) $R_{g}$ plot exhibiting ANXA2 and ANXA2-curcumin complex compactness over 100 ns simulation, (B) SASA plot exhibiting ANXA2 and ANXA2-curcumin complex solvent accessibility area over 100 ns simulation. Probability distribution plot of (C) $R_{g}$ and (D) SASA.

## Abbreviations

OC: ovarian cancer; APO, Apolipoprotein; ANX, Annexin; PCD, programmed cell death; CT, computed tomography; CA-125, cancer antigen-125; TF, transcription factor; IBD, inflammatory bowel disease; LC, lung cancer; HGNC, HUGO Gene Nomenclature Committee; GEPIA, Gene Expression Profiling Interactive Analysis; mRNA, Messenger RNA; TCGA, The Cancer Genome Atlas; GTEx, Genotype-Tissue Expression; FC, fold change; KM, Kaplan–Meier; OS, overall survival; CNA, copy number alteration; GO, gene ontology; BP, biological process; CC, cellular compartment; MF, molecular function; PDB, protein data bank; RCSB, Research Collaboratory for Structural Bioinformatics; RO5, Lipinski’s rule of 5; LI, Ligand efficiency; ADMET, absorption, distribution, metabolism, excretion, and toxicity; MD, molecular dynamics; CGenFF, CHARMM General Force Field; NVT, constant- temperature, constant-volume ensemble; NPT, constant-temperature, constant-pressure; aa, amino acid; RMSD, root mean square deviation; RMSF, root mean square fluctuation; $R_{g}$, radius of gyration; SASA, Solvent accessible surface area; DGE, differential gene expression; GF, growth factor; ApoE, Apolipoprotein E; CAD, coronary artery disease; TME, tumor microenvironment; GI, genomic instability; GC, germ cell; TA, tumor angiogenesis; RCC, renal cell carcinoma; TNBC, triple-negative breast cancer; CID, chronic inflammatory disease; CP, cell proliferation; GED, gene expression data; OS, oxidative stress; H, hydrogen; PDF, probability distribution function; RA, Rheumatoid Arthritis; FF, force field; EMT, epithelial–mesenchymal transition; TPSA, topological polar surface area; MMP, matrix metalloproteinase; EGFR, epidermal growth factor receptor.
