# Supplementary material for: Exploring the Prognostic, Mutational and Therapeutic Potential of ANXA2 in Ovarian Cancer via Multi-Omics and In Silico Approach
Source: Biology (Basel). 2026 Mar 25;15(7):523. doi: 10.3390/biology15070523 (PMC13071996; doi:10.3390/biology15070523)
Supplement: Supplementary file 1 [file biology-15-00523-s001.zip › biology-4102163-supplementary.pdf]

<u>**Supplementary Data**</u>

Exploring the Prognostic, Mutational and Therapeutic Potential of ANXA2 in Ovarian Cancer via Multi-Omics and In-Silico Approach

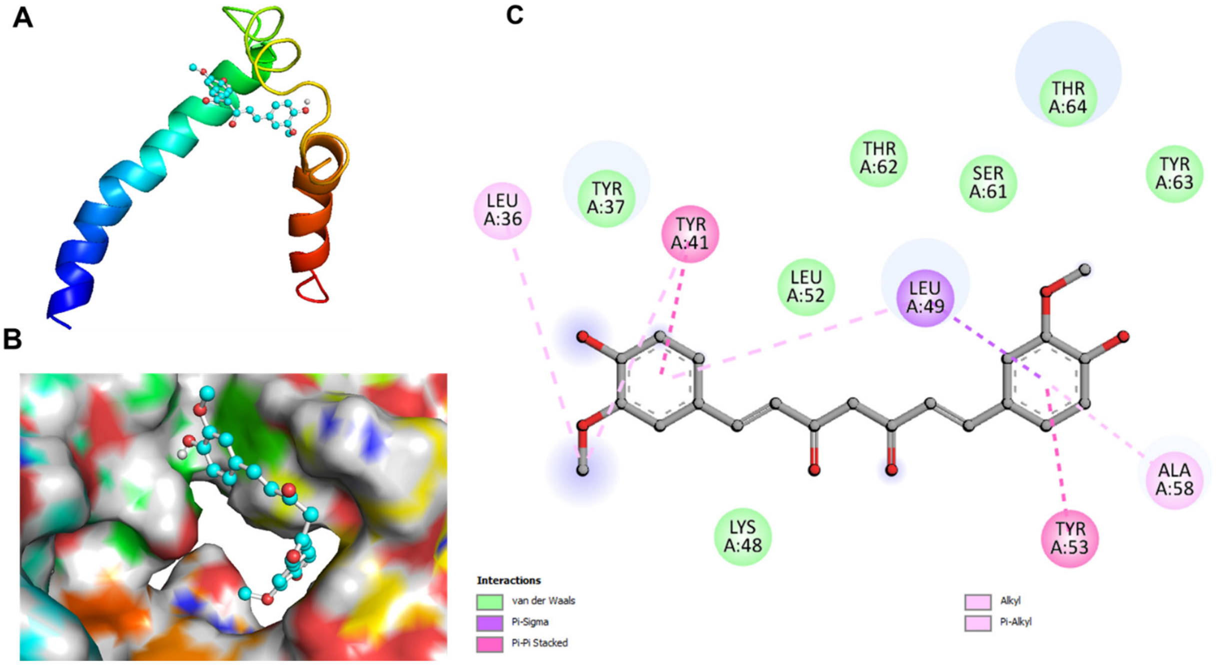

Figure S1. *Representation of molecular docking result.* ***(A)*** *cartoon view of APOC2 in-complex with Curcumin.* ***(B)*** *Surface view of APOC2 in-complex with Curcumin.* ***(C)*** *2D view of interacting residues of APOC2 with Curcumin.*

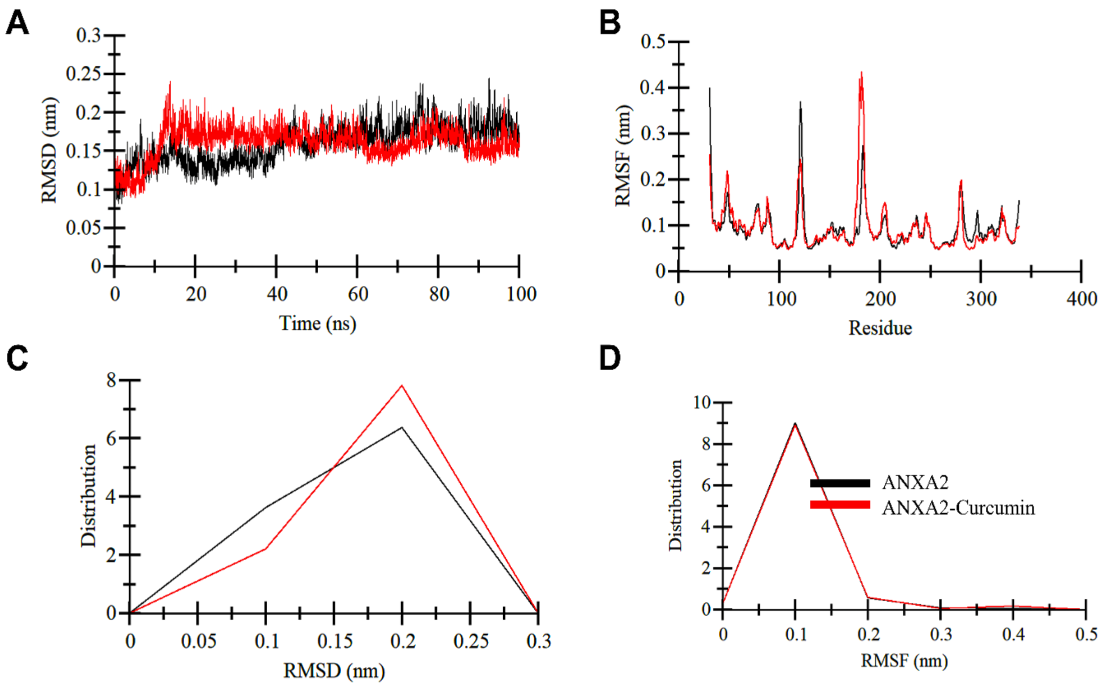

Figure S2. ***(A)*** *RMSD plot exhibiting ANXA2 and ANXA2-curcumin complex deviation over* 100 *ns simulation.* ***(B)*** *RMSF plot exhibiting ANXA2 and ANXA2-curcumin complex fluctuation over 100 ns simulation. Probability distribution plot of* ***(C)*** *RMSD and* ***(D)*** *RMSF.*

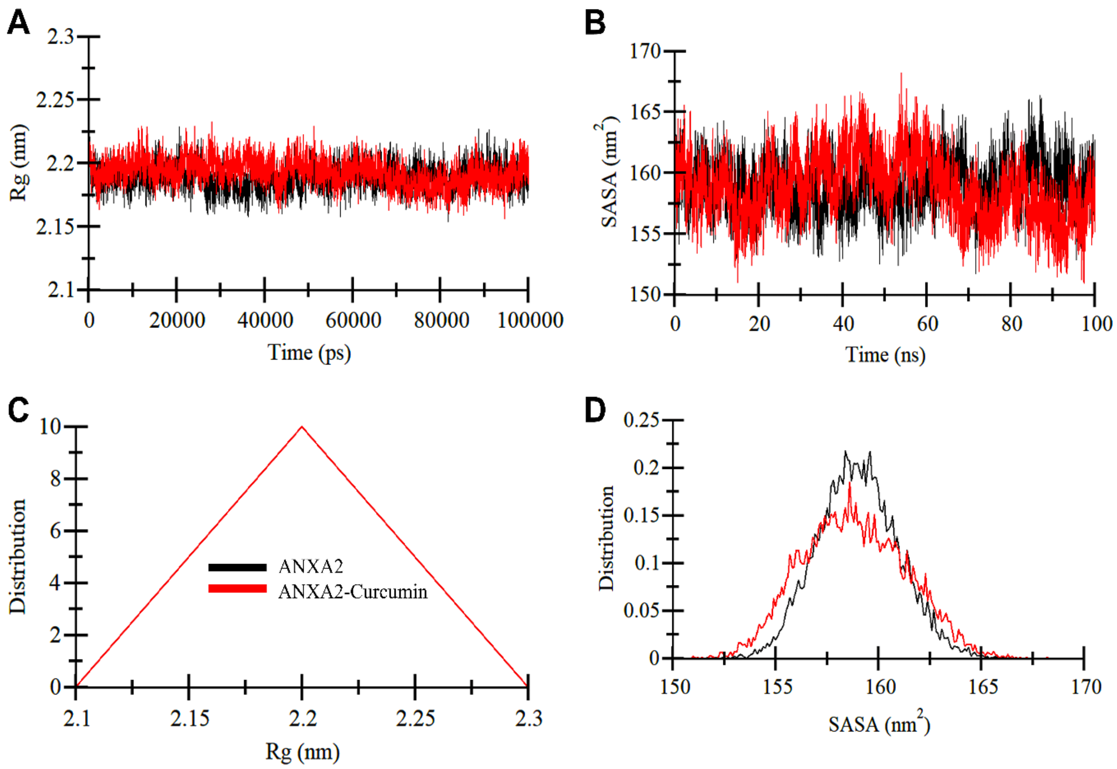

Figure S3. ***(A)*** *R_g plot exhibiting ANXA2 and ANXA2-curcumin complex compactness over 100 ns simulation,* ***(B)*** *SASA plot exhibiting ANXA2 and ANXA2-curcumin complex solvent accessibility area over 100 ns simulation. Probability distribution plot of* ***(C)*** *R_g and* ***(D)*** *SASA.*
